# Organizational determinants of information transfer in palliative care teams: A structural equation modeling approach

**DOI:** 10.1371/journal.pone.0252637

**Published:** 2021-06-03

**Authors:** Reka Schweighoffer, Richard Blaese, Brigitte Liebig

**Affiliations:** 1 Department of Psychology, University of Basel, Basel, Switzerland; 2 School of Applied Psychology, University of Applied Sciences Northwestern Switzerland, Olten, Switzerland; 3 Department of Sociology, University of Basel, Basel, Switzerland; University of Birmingham, UNITED KINGDOM

## Abstract

Several organizational factors facilitate or hinder information transfer in palliative care teams. According to past research, organizational factors that reduce information transfer include the inconsistent use of shared electronic patient files, frequent changes of healthcare staff, a lack of opportunities for personal exchange, and a lack of evaluation of collaborative processes. Insufficient information sharing between professionals can negatively impact patient safety, whereas studies have shown that some organizational factors improve collaboration between professionals and thus contribute to improved patient outcomes. The main purpose of this study is thus to investigate whether, and if so how, organizational factors contribute to successful information exchange in palliative care teams in Switzerland, while also accounting for the different care contexts of primary and specialized palliative care. A nationwide survey was aimed at medical professionals working in palliative care. In total, 379 participants (mean age = 49.8 years, SD = 10.3) were included in this study. Two main outcome variables were examined: healthcare providers’ satisfaction with information transfer in their team and their overall satisfaction with communication in their team. Hypotheses were tested by employing structural equation modeling. Findings revealed that the strongest predictors for effective information transfer in palliative care teams were sufficient opportunities for face-to-face meetings and supervision alongside feedback tools to improve collaborative practices and the application of guidelines and standards for collaboration. Face-to-face meetings were an even greater contributor to information transfer in specialized settings, whereas sharing the same work-based values with colleagues was considered more important in primary settings. Results from this study contribute to the existing literature elucidating how information transfer is facilitated in the field of palliative care. If proposed measures are implemented, this could possibly improve patient outcomes in palliative care. Furthermore, the findings can be useful for healthcare organizations and associations to make more efficient resource allocation decisions with the aim to optimize information transfer within the workforce.

## Introduction

The topic of interprofessional collaboration in healthcare has received considerable attention in recent decades and has specifically gained importance in the interdisciplinary field of palliative care (PC). Due to the broad spectrum of PC patients’ needs, successful PC delivery relies on efficient collaboration between medical doctors, nurses, and a wide range of support services within and across different institutions and settings [1]. Interprofessional collaboration in healthcare is described as the collaboration between at least two professionals with differing specializations who work interdependently of each other, fulfill specific roles, and share the same work-related, patient-centered goals [2]. The multiple advantages of successful interdisciplinary collaboration in PC are confirmed by recent reviews, which report of increased patient satisfaction with health services and optimized referral processes, as well as cost reductions and improved symptom management in patients [3–5]. In particular, research highlights the importance of seamless information transfer between and within healthcare professionals as a major contributor to fruitful collaboration, which is the main focus of this research paper [6].

### Significance of organizational enablers on information transfer in teams

Palliative care teams can be described generally as complex, flexible, yet adaptive structures that shape the team, its members, and its environment [7]. Bainbridge et al. (2010) proposed a comprehensive input-process-output (I-P-O) model to evaluate PC services. This framework postulates that collaboration in PC teams is comprised of three main elements: systemic, process of care, and patient outcome-related determinants [8]. Organizational aspects of collaboration, which are part of the “process of care,” entail team resources and administrative support, tools that facilitate or regulate information transfer and coordination, and shared team values [8]. In a review of the determinants of successful collaboration, San Martin and colleagues suggested that systemic determinants (e.g. the structural embeddedness of care teams) have received more attention in collaboration research than organizational aspects [9]. In PC specifically, a large knowledge gap exists regarding what organizational and care-process related factors promote an efficient collaboration in terms of improved information transfer. However, Bainbridge et al. (2010) argues that in the case of palliative care, satisfactory patient outcomes can only be achieved via efficient information transfer and satisfactory team communication. Moreover, with the rise of new concepts for interprofessional communication in healthcare, an evaluation of key mechanisms that foster collaboration on an intraorganizational level in PC is needed more now than ever before [10].

There are several organizational barriers to information transfer. Often originating from a lack of structural resources and time pressure, organizational factors that can hinder information transfer in PC teams include the lack of standardized guidelines for collaboration, the inconsistent use of shared electronic patient files, and the lack of opportunities for personal exchange and feedback through meetings or supervisions [8, 9].

The primary objective of this study is to test the influence of select organizational variables on the perceived quality of information exchange of PC providers. Moreover, this study exploratively assesses how the quality of information transfer affects PC providers’ perceived satisfaction with collaboration, as well as their satisfaction with job-related tasks.

The methods section in this paper is presented in three parts. First, the paper will examine the organizational variables that facilitate or hinder information exchange in the study sample of Swiss PC providers. Second, the paper investigates whether information transfer affects PC providers’ satisfaction with communication, and consequently, their satisfaction with job-related tasks. Third, the paper investigates if certain organizational determinants for information transfer are context dependent. For this purpose, the two settings of primary palliative care (PPC) and specialized care (SPC, test for moderation) are distinguished.

### The impact of organizational variables on information transfer

The development of interprofessional collaboration in healthcare has been shown to vastly benefit from the formalization of rules and procedures [10, 11]. Existing research literature suggests that the application of standardized procedures leads to improved information exchange in healthcare teams, as well as to enhanced communication with patients themselves [9–11].

Establishing standardized procedures is best achieved by the dissemination and application of guidelines and standards for best practices for interprofessional collaboration [8]. The use of best-practice guidelines and standards for collaboration (e.g. standardized communication protocols), in turn, can result in a more balanced share of role responsibilities between providers, which also facilitates information exchange [12]. Furthermore, opportunities for formal and informal face-to-face meetings, group discussions, and roundtables have been highlighted as facilitators for information exchange [10, 13]. Regular face-to-face contact of the team members fosters team cohesion and trust in healthcare teams and helps to build lasting care networks [10, 13]. E-tools in the form of electronic health records allow members to easily share and update patient information and are widely used in Switzerland by specialized PC facilities, such as hospitals and hospices [14]. According to the World Health Organization (2019), if well designed and implemented, electronic patient files improve information transfer and facilitate handovers between healthcare providers [15]. However, it remains uncertain whether electronic tools to share patient information are perceived as helpful by providers in Swiss palliative care provision. In addition to providing formalized channels for information exchange and opportunities for face-to face meetings, the literature has pointed to the importance of the management and coordination of processes by predestined administrative personnel, such as case managers [16–18]. Case management (CM) is a widely used term for mostly administrative aspects of care, consisting largely of planning, implementing, coordinating, and monitoring of service needs of healthcare providers, patients, and patients’ families [17]. Some research has identified positive effects of the presence of CM on improved information exchange, which in turn, improves quality of patient care in PC [16, 18]. However, if and how case managers facilitate information transfer in PC teams still remains unclear [17]. The researchers hypothesize that due to its coordinative nature, the presence of a CM in the immediate work environment improves information transfer and increases PC providers’ satisfaction with communication.

With respect to the healthcare setting, frequent transitions of healthcare providers hinder information flow within the team [19]. This led us to hypothesize that frequent changes in PC staff would impair information transfer. Finally, opportunities to provide feedback and evaluate ongoing work processes have been cited as an essential factor to foster information transfer. Research implies that only by generating opportunities to improve collaborative processes via feedback rounds, it is possible to maintain successful interpersonal networks at the workplace possible over time [20].

Thus, based on the theoretical framework of Bainbridge et al. 2010, the primary objective of this study is to examine the influence of certain organizational variables on the perceived quality of information exchange of Swiss PC providers. The following hypotheses concerning organizational determinants were tested in the first part of this study:

**H1a:** The availability of internal guidelines and standards increases information exchange within the team.
**H1b:** The use of internal guidelines and standards increases information exchange within the team.
**H1c:** The opportunity for face-to-face meetings (e.g. in the context of meetings, roundtables, and supervisions) increases information exchange.
**H1d:** The use of electronic tools to manage patient files increases information exchange within the team.
**H1e:** The regular evaluation of work processes with quality circles or feedback rounds increases information exchange within the team.
**H1f:** The presence of a case manager in the immediate work environment increases information exchange within the team.
**H1g:** Frequent changes of caregivers in a team reduces general information exchange in the team.

### The impact of information transfer on providers’ satisfaction with collaboration

This part of the study investigates what factors affect PC providers’ satisfaction with communication in PC teams. Ultimately, team communication in healthcare is more than just accurate information transmission. Multidisciplinary PC teams are socially constructed groups that operate at the intersection of multiple institutional and professional cultures [21]. PC professionals are more likely to develop mutual respect and a trusting working relationship if they share certain professional standards and values regarding patient care [20, 22, 23] The importance of shared values and standards for teamwork is also emphasized in Bainbridge et al.’s (2010) I-P-O model under “process of care.”

Moreover, healthcare research indicates that an open communication culture is facilitated by a clear delineation of roles and tasks among team members, alongside collective risk-taking [20, 24]. Therefore, researchers have hypothesized that a clear division of responsibility, as well as shared values between fellow PC providers, improves HCPs satisfaction with collaboration in their respective work teams.

The study also explores how satisfaction with communication affects providers’ satisfaction with job-related tasks. Impaired communication in PC teams can lead to increased misunderstandings at the workplace, which can trigger disputes within the workforce and lower job satisfaction for providers [25]. PC providers’ satisfaction with job-related tasks, in turn, positively impacts patient safety, as healthcare providers who enjoy their work tend to show better clinical performance and remain longer in the same healthcare team [24, 26]. Since the satisfaction of team members is linked to staff retention, this is a critical element for team functioning, as well as a major predictor for good healthcare provision [24, 26].

In order to investigate HCPs satisfaction with communication, the following hypotheses were tested:

**H2a:** The extent of information exchange in the team predicts providers’ satisfaction with communication.
**H2b:** A clear division of roles within the team increases providers’ satisfaction with communication.
**H2c:** If providers share the same values, this increases their satisfaction with communication.
**H2d:** Providers’ satisfaction with communication increases their satisfaction with work-related tasks.

### The context of primary and specialized palliative care as a moderating factor

This study investigates whether the importance of certain organizational variables for information transfer is moderated by the context of two different PC-settings, namely primary palliative care (PPC) and specialized palliative care (SPC), which are distinguished by the patients’ current condition [1]. Patients in primary care (Group A) require basic PC and are mostly treated in retirement homes or home-care settings. Patients who receive specialized palliative care (Group B) receive complex medical and psychosocial PC that is provided at acute-care hospitals or hospices, as well as by specialist mobile palliative care teams [1].

Especially in PPC, general practitioners (GPs) and nurses face limited time and financial reimbursement for collaborative activities, which can result in gaps in information sharing and, consequently, in care shortages [27]. Moreover, GPs often work in private practices while nurses are organized in local or private nursing-groups. This can lead to spatial fragmentation of the PPC care team and represent a barrier to efficient information transfer [27, 28]. Therefore, PC providers in PPC sharing the same patient-centered values and ideals might be especially important so that, despite spatial barriers, healthcare providers feel motivated to share valuable information since they feel personally committed to their coworkers [23].

A greater degree of institutionalization can be expected in SPC, where different healthcare providers work together in proximity and where collaboration is often governed by existing guidelines and standards [1, 8]. Looking at the sphere of SPC, e-tools are likely used to share patient files and therefore contribute more to information exchange. Furthermore, routine face-to-face meetings and supervisions may be more important in the context of SPC, where, due to the more complex patients, more rapid information exchanged is required [29]. Based on profound differences in the two care contexts of PPC and SPC, the third part of the study aims to identify context-dependent organizational prerequisites for successful information transfer, and tests the following hypotheses:

**H3a:** Colleagues who share the same values report higher satisfaction with communication, especially in the setting of primary palliative care.
**H3b:** The use of e-tools to share patient files is expected to play a stronger role for information transfer in specialized palliative care.
**H3c:** Opportunities for face-to-face exchanges in the form of meetings and supervisions are expected to contribute to better information sharing in both settings, but especially in specialized palliative care.

**Fig 1:**
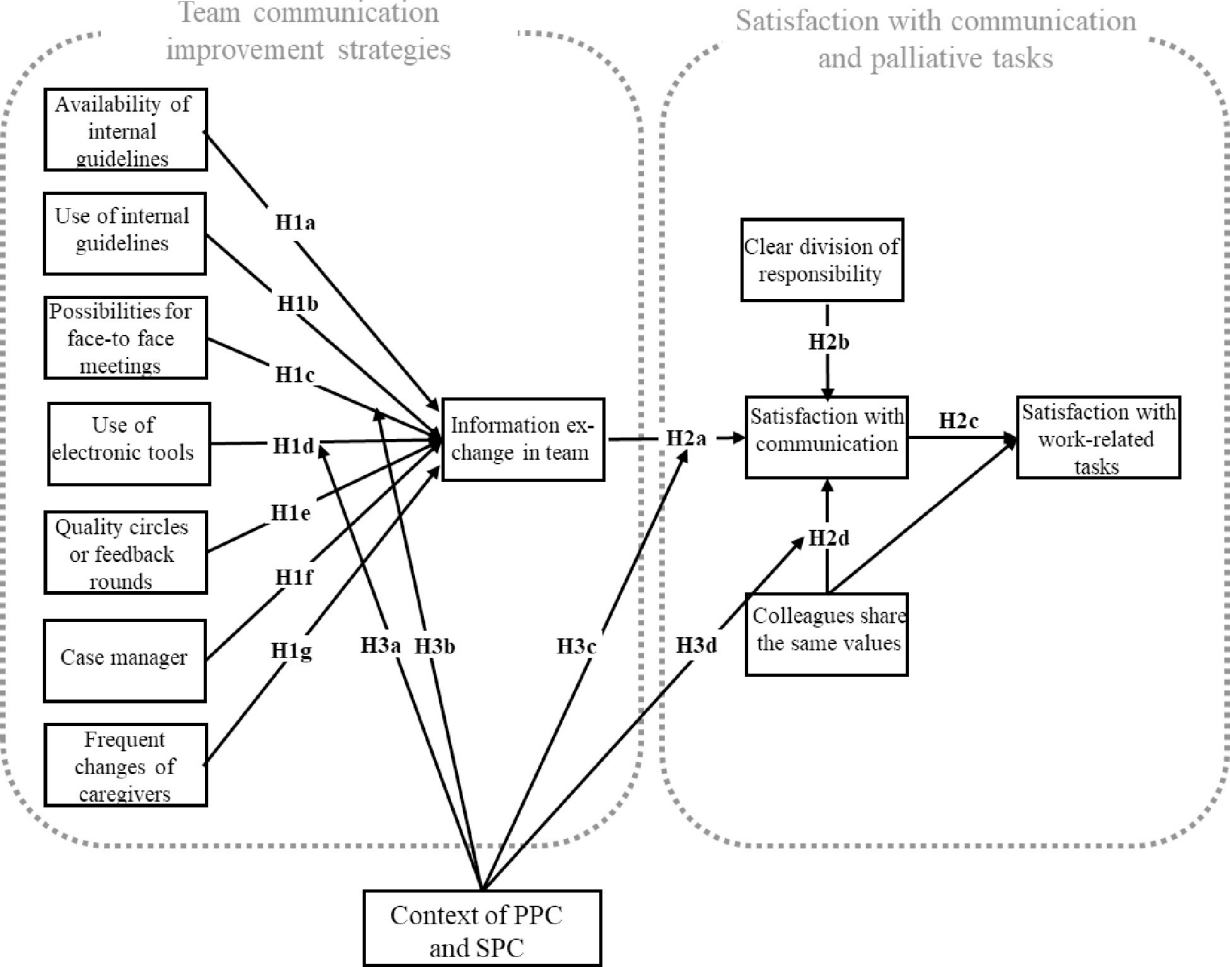
Theoretical model. Hypothesized predictors of information exchange in the team and hypothesized additive and interactive effects of information exchange in the team on satisfaction with communication and satisfaction with work-related tasks (after Bainbridge et al., 2010).

To date, explorations of the organizational factors that improve information transfer and the dissemination of patient information is rare in PC-related contexts. Therefore, the first and main objective of this study is to test the influence of the organizational variables mentioned above on the perceived quality of information exchange and the dissemination of patient information. Hypothesized predictors of information exchange in the team and hypothesized additive and interactive effects of information exchange in the team on satisfaction with communication and satisfaction with work-related tasks are depicted in Fig 1.

## Methods

To examine associations of organizational factors and information transfer on multiple levels, the study uses structural equation modeling [SEM, 30]. This study is the first to use SEM to show in detail what levers improve information transfer at an organizational level while also accounting for the two different settings of PC provision.

### Participants

The survey was aimed at healthcare professionals, primarily medical doctors and nurses, who were active in palliative care provision in Switzerland in 2017. The data collection via email was performed between September 19 and November 30, 2018. Three rounds of reminders, including informed consent, were sent out the following month. In Switzerland, we estimate the total number of palliative care providers, including GPs who regularly treat palliative care patients, at around 4500. We used a sample gathered from a wide range of healthcare units, including acute-care hospitals, nursing associations, hospices and nursing homes, which allows our research design to be informative without relying on transnational data. A total number of 1,111 healthcare providers who are actively involved in palliative care provision took part in the online study (f = 64.7%, m = 14.3%, mean age = 50.9 years, SD = 10.3). At around 24.5%, the response rate of this study can therefore be considered representative for the Swiss healthcare context.

In order to contact medical doctors and nurses, a two-step recruiting approach was carried out by identifying organizations of interest, which then recruited their employed or associated healthcare providers to complete the survey. The anonymity of responders was ensured at all times and the study data was handled in accordance with the Swiss law governing the use of scientific data.

### Measures

The survey items were adapted from Bainbridge et al.’s (2010) tool for evaluation of healthcare provision [8], and supplemented with items drawn from the Index of Interdisciplinary Collaboration from Bronstein (IIC)– 42 [31] and basic demographic data of participants (age, gender, occupational field, institutional affiliation). The items of the questionnaire were translated by professionals in a multistage process into German, French, and Italian versions. The final questionnaire was composed in an online survey provider and required 25–30 minutes to complete.

### Dependent variables

Two items were selected as main outcome variables.

#### PC providers’ satisfaction with information transfer in their PC team

In order to assess information transfer in their care teams, PC providers were asked to evaluate the information exchange with their immediate team members (6-point Likert scale: “very good” to “very bad”).

#### PC providers’ satisfaction with communication

To assess the satisfaction with overall communication in providers’ immediate work environments, the following item was selected: The communication within our organization/our institute is good (6-point Likert scale: “does not apply at all” to “fully applies”).

### Independent variables

The following seven predictor variables for information transfer were measured: (1) the availability and use of internal guidelines and standards for collaboration in the providers’ immediate work environment (two dichotomy items: 0 = no, 1 = yes), (2) if a clear share of responsibility was present in the immediate work environment (4-point Likert scale: “yes,” “rather yes,” “rather no,” to “no”), (3) if regular opportunities for face-to-face meetings were present, (4) whether or not the team used electronic tools to manage patient files, (5) whether or not work processes were regularly evaluated with quality circles or feedback rounds, (6) whether or not PC providers had a case manager in their immediate work environment (all dichotomy, dummy-coded items: 0 = no, 1 = yes), (7) and whether or not there were frequent changes of caregivers in the immediate work environment (4-point Likert scale: “yes”, “rather yes”, “rather no”, and, “no”).

The following covariates were included in the analysis to control for gender (0 = male; 1 = female), age, position (leading vs. no leading position), socio-geographic workplace (1 = large city, 5 = small village in rural area), job description (nurse, medical doctor), and additional training in palliative care.

### Statistical analyses

All hypotheses were tested using a structural equation model via the SEM function of the package ‘Lavaan’ (latent variable analysis, version 0.6–4, in R: Development Core Team 2012) [32, 33]. This method allows researchers to test path models, including latent variables that are not affected by measurement error. The following fit indices were evaluated according to standards in social science after Kline et. al (2015): chi-square (*X*^2^), Comparative fit Index *(CFI*) [for testing the overall fit], root mean square of approximation *(RMSEA*) [for model complexity], and *Tucker-Lewis Index (TLI)* [34]. According to best practice, a good model fit is considered by p value for the model <0.05, RMSEA <0.06, CFI and TLI ≥ 0.90 [34]. Missing values were handled according to the method of listwise deletion.

## Results

### Sample characteristics

In total, 379 participants, aged between 24 and 76 years (Mean = 49.8 years, SD = 10,3) were included in this study. The detailed sample description is summarized in Table 1.

**Table 1:** Baseline demographic of the study sample of n = 379 palliative caregivers.

	PPC (n = 229)	SPC (n = 150)	Overall (n = 379)
**Age**			
Mean (SD)	50.7 (9.86)	48.4 (10.9)	49.8 (10.3)
Median [Min, Max]	53.0 [25.0, 75.0]	50.0 [24.0, 76.0]	52.0 [24.0, 76.0]
**Gender**			
Male	31 (13.5%)	36 (24.0%)	67 (17.7%)
Female	198 (86.5%)	114 (76.0%)	312 (82.3%)
**Function**			
Nurses	196 (85.6%)	111 (74.0%)	307 (81.0%)
Medical doctors	33 (14.4%)	39 (26.0%)	72 (19.0%)
**Workplace demographics**			
Larger city	69 (30.1%)	96 (64.0%)	165 (43.5%)
Other	160 (69.9%)	54 (36.0%)	214 (56.5%)
**Additional training**			
None	70 (30.6%)	39 (26.0%)	109 (28.8%
Yes	159 (69.4%)	111 (74.0%)	270 (71.2%
**E-Tool to share patient files**			
No	35 (15.3%)	13 (8.7%)	48 (12.7%
Yes	194 (84.7%)	137 (91.3%)	331 (87.3%)
**Case Manager**			
No	182 (79.5%)	93 (62.0%)	275 (72.6%)
Yes	47 (20.5%)	57 (38.0%)	104 (27.4%)
**Guidelines for collaboration available**			
No	17 (7.4%)	2 (1.3%)	19 (5.0%)
Yes	212 (92.6%)	148 (98.7%)	360 (95.0%)
**Application of these guidelines**			
No	29 (12.7%)	11 (7.3%)	40 (10.6%)
Yes	200 (87.3%)	139 (92.7%)	339 (89.4%

### Correlation coefficients

Standard deviations (SD) and zero-order correlations are provided in Table 2. Aligned with *a priori* expectations, the majority of the organizational variables were significantly correlated to information exchange, as well as to providers’ satisfaction with communication. Especially regarding the opportunities for face-to-face meetings in the context of meetings, round-tables, and supervisions (rho = 0.57, p <0.001), colleagues who share the same values (rho = 0.44, p <0.001), and the use of feedback-tools (rho = 0.31, p <0.001) were positively correlated to the information exchange within the team.

**Table 2:** Standard deviations and correlations with confidence intervals.

	1	2	3	4	5	6	7	8	9	10	11	12	13	14	15
1 Information exchange in the team	1														
2 Guidelines available	0.15**	1													
3 Application of these guidelines	0.26***	0.59***	1												
4 E-Tools to share patient files	0.02	0.09	0.05	1											
5 Case Manager	0.15**	0.06	0.10	0.04	1										
6 Feedback Tools	0.32***	0.21***	0.25***	-0.03	0.17**	1									
7 Opportunities for face to face meetings	0.58***	0.22***	0.23***	-0.00	0.10*	0.35***	1								
8 Frequent changes of caregivers	-0.31***	-0.11*	-0.11*	0.02	-0.04	-0.15**	-0.28***	1							
9 Good division of responsibility	0.28***	0.21***	0.23***	0.09	0.15**	0.24***	0.25***	-0.22***	1						
10 Colleagues share the same values	0.44***	0.07	0.24***	0.01	0.03	0.21***	0.28***	-0.28***	0.25***	1					
11 Satisfaction with communication	0.43***	0.11*	0.21***	-0.06	0.04	0.19***	0.32***	-0.31***	0.26***	0.46***	1				
12 Satisfaction with work-related tasks	0.29***	0.04	0.15**	-0.06	0.01	0.14**	0.20***	-0.18***	0.18***	0.47***	0.40***	1			
13 Workplace demographics	0.08	0.08	0.06	0.05	0.19***	0.07	0.16**	0.07	-0.02	0.06	0.03	0.04	1		
14 Additional training	-0.03	-0.01	0.05	-0.05	-0.01	0.02	-0.03	0.12*	0.04	-0.03	-0.05	0.10*	-0.10	1	
15 Gender	-0.09	0.05	0.07	-0.03	0.01	0.05	-0.13*	0.14**	-0.08	0.08	-0.03	0.05	-0.05	0.12*	1

Pearson correlation coefficient (1-tailed),

* indicates p <0.05.

** indicates p <0.01.

*** indicates p < 0.001. Correlations of binary variables should be interpreted with care.

### Structural equation modeling

#### Dependent variable: Information exchange in the team

Following best statistical practices, we report the measurement model on the full sample of n = 379 [33]. The researchers first tested the hypothesized model (Fig 1) including control variables. This model achieved a good fit (*X*^2^ [30] = 57.1,*p* = 0.002; *CFI* = 0.94; *RMSEA* = 0.49; *TLI* = 0.91) and accounted for 39% of the variance in information exchange in the team, 26% of variance in satisfaction with communication, and 29% of variance in satisfaction with work-related tasks.

#### H1a

Internal guidelines and standards are relevant to information exchange; thus, their presence should improve information exchange within the team. We found little evidence in support of this hypothesis (β = -0.09, p = 0.08).

#### H1b

The use of those available internal guidelines and standards significantly explained the increase in information exchange within the team (β = 0.15, p<0.01).

#### H1c

The opportunity for face-to-face meetings (e.g. in the context of meetings, roundtables, and supervisions) significantly explained the increase of information exchange within the team (β = 0.48, p<0.001).

#### H1d

The use of electronic tools to manage patient files was not significantly correlated to an increase in information exchange within the team (β = 0.03, p = 0.50).

#### H1e

The regular evaluation of work processes with quality circles or feedback rounds predicted information exchange within the team (β = 0.10, p<0.041).

#### H1f

The presence of a case manager in the immediate work environment results did not significantly explain changes in information exchange within the team (β = 0.07, p = 0.09).

#### H1g

Frequent changes of caregivers in a team indeed predicted general information exchange in teams negatively (β = -0.15, p<0.001).

**Fig 2:**
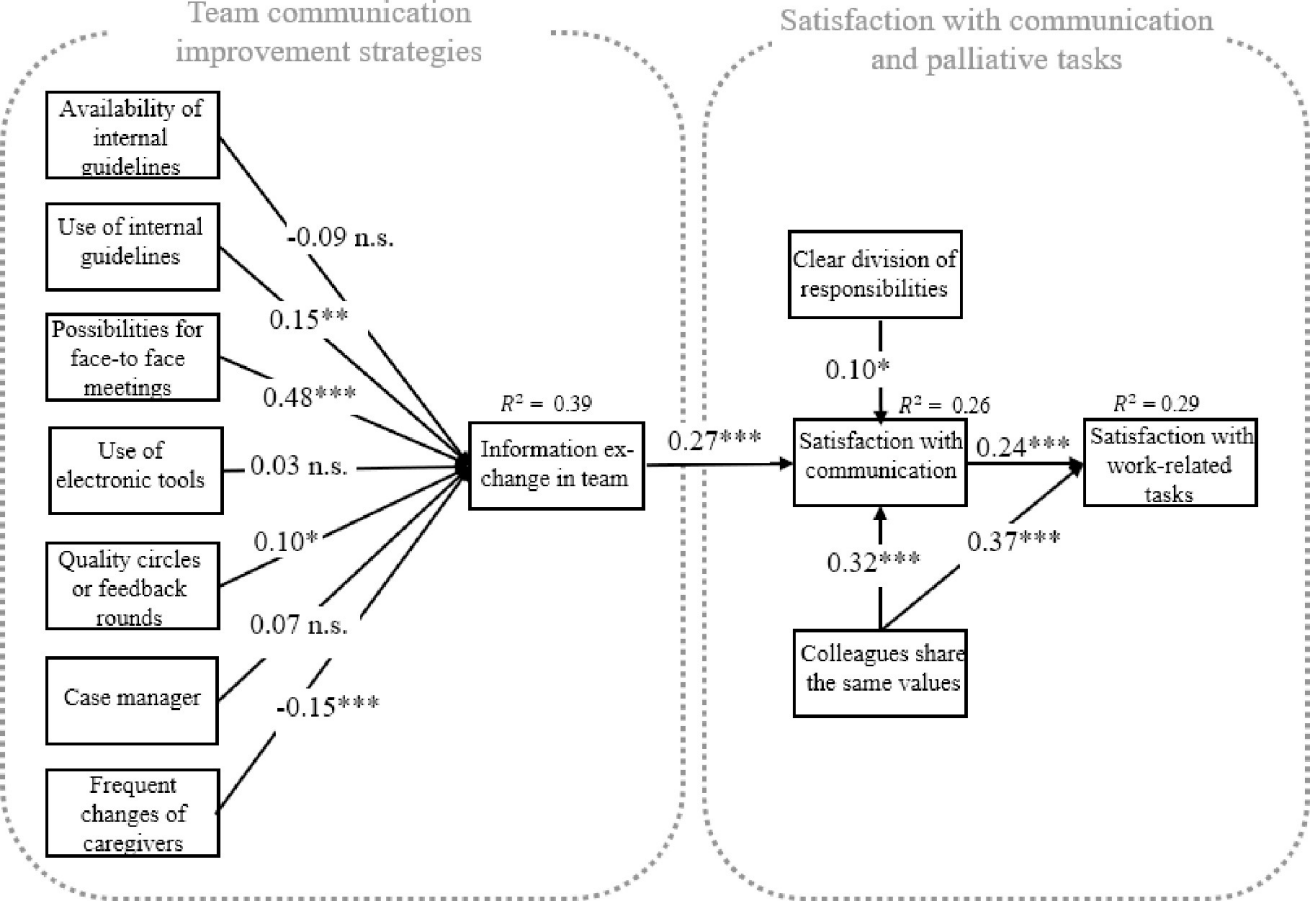
Empirical model. * indicates p < 0.05 ** indicates p < 0.01 *** indicates p < 0.005. Standardized effects are given. All effects are controlled for position (lead/no lead), type of caregiver (context), place of work (city vs. countryside), function (job description), additional training, and gender; n = 379.

By applying a structural equation model, there was no support for hypotheses H1a, H1d, and H1f (for an overview, see Table 4). The empirical model is depicted in Fig 2.

### The impact of information transfer on provider’s satisfaction with collaboration

#### H2a

The more frequently information is exchanged in the team, the more satisfied are care providers with communication (β = 0.27, p<0.001).

#### H2b

A clear division of responsibility within the team positively predicted providers’ satisfaction with communications (β = 0.10, p<0.032).

#### H2c

When colleagues felt that they shared the same values, this was positively associated with their satisfaction with communication (β = 0.32, p < 0.001) as well as their satisfaction with work-related tasks (β = 0.37, p < 0.001).

Exploratively, this study investigated the extent to which providers’ satisfaction with their communication affects their satisfaction with job-related tasks (H2d). Indeed, the results provide considerable evidence that providers’ satisfaction with communication positively predicts their satisfaction with work-related tasks (β = 0.24, p<0.001). Little support for hypotheses H2a-H2d were found (see Table 4).

### Moderating effect of care giving context of PPC vs. SPC: H3a-H3c

In order to test the moderating effect of care-giving context of primary care versus specialized care on select organizational factors, cross-group structural equalization modeling was employed. In both groups of PPC (n = 229) versus SPC (n = 150), the model explained a considerable amount of variance of information exchange in the team (34%, 46%), as well as providers’ satisfaction with work related tasks (31%, 28%).

#### H3a

Unsurprisingly, colleagues who share the same values reported higher satisfaction with communication in their team. Within PPC (β = 0.42, p < 0.001), individuals who reported sharing the same care-based values and ideals indeed showed higher predictive scores of satisfaction with communication as compared to SPC (β = 0.14, p = 0.06).

#### H3b

The use of e-tools to share patient files is expected to play a stronger role for information exchange in SPC. The use of e-tools to exchange patient records showed no significant effect on information exchange in teams, neither in PPC (β = 0.01, p = 0.85) nor in SPC (β = 0.06, p = 0.31).

#### H3c

Opportunities for interprofessional exchange, such as face-to-face meetings, are expected to contribute to greater information sharing in SPC. Indeed, interprofessional exchange in the form of face-to-face meetings and supervisions had a strong effect for the setting of SPC (β = 0.60, p < 0.001), compared to PPC (β = 0.40, p < 0.001).

A moderation analysis including X^2^ difference tests were performed to test whether the group differences of the paths are statistically significant. First, the researchers tested for measurement invariance across the groups by comparing the unconstrained multi-group model with a constrained multi-group model where the respective factor loadings and measurement intercepts were set equal for both groups. A difference test on χ^2^ showed no difference between the two models (Δχ^2^ [3] = 1.85, p = 0.61), indicating measure invariance across both groups. Second, researchers tested the unconstrained model against models where one of the paths was set equal across both groups (Table 3). A moderation effect of the care context was found for the relationship between H3a, sharing the same values with colleagues and satisfaction with communication (Δχ^2^[1] = 9.6, p < 0.001), and H3c, opportunities for interprofessional exchange, such as face-to-face meetings and supervisions, and satisfaction with communication (Δχ^2^[1] = 7.05, p < 0.01). Little evidence was found for a moderating effect of the care context in the relationship between H3b, e-tools to share patient files, and the exchange of patient information (Δχ^2^[1] = 0.41, p = 0.50). Statistical support for hypotheses H3a and H3c was found, whereas H3b had little support (see Table 4).

**Table 3:** Fit indices and χ2 difference test for moderation effect of context.

Models	χ2	df	CFI	RMSEA	Δχ2	Δdf
Unconstrained model	76.98	56	0.95	0.044		
*H3a) Colleagues share same values* -> satisfaction with communication; set equal across groups	86.60	57	0.93	0.052	9.61**	1
*H3b)* Use of e-tools -> info exchange in team; set equal across groups	77.44	57	0.95	0.044	0.46	1
*H3c)* Opportunities for face-to face meetings -> info exchange in the team; set equal across groups	84.03	57	0.94	0.050	7.05**	1
*Amount of i*nformation exchange in the team -> providers`satisfaction with communication; set equal across groups	82.50	57	0.94	0.048	5.50*	1

Note: * indicates p < 0.05

** indicates p < 0.01

*** indicates p <0.001.

**Table 4:** All hypotheses at one glance.

Nr	Hypotheses	Value	True/False
H1a	The availability of internal guidelines and standards increases information exchange within the team.	*β* = - 0.09 n.s.	Not confirmed
H1b	The use of those available internal guidelines and standards increases information exchange within the team.	*β* = 0.15**	Confirmed
H1c	The opportunity for face-to-face meetings (e.g. in the context of meetings, roundtables and supervisions) increases information exchange.	*β* = 0.48***	Confirmed
H1d	The use of electronic tools to manage patient files increases information exchange within the team and fosters continuous exchange of patient information.	*β* = 0.03 n.s.	Not confirmed
H1e	The regular evaluation of work processes with quality circles or feedback rounds increases information exchange within the team.	*β* = 0.10*	Confirmed
H1f	The presence of a case manager in the immediate work environment results in increased information exchange within the team	*β* = 0.07 n.s.	Not confirmed
H1g	Frequent changes of caregivers in a team hinder general information exchange.	*β* = -0.15***	Confirmed
H2a	The extent of information exchange in the team predicts providers`satisfaction with communication.	*β* = 0.27***	Confirmed
H2b	A clear division of responsibility within the team increases information exchange within the team and fosters continuous exchange of patient information.	*β* = 0.10*	Confirmed
H2c	Sharing the same values increases providers`satisfaction with communication	*β* = 0.32***	Confirmed
H2d	Provider`s satisfaction with communications affects their satisfaction with work-related tasks.	*β* = 0.24***	Confirmed
H3a	Colleagues who share the same values report higher satisfaction with communication especially in the primary palliative care setting.	*(Δχ2[1] = 9*.*61*, *p<0*.*01)*	Confirmed
H3b	The use of E-tools to share patient files is expected to play a stronger role for information transfer and satisfaction with communication in specialized settings.	*(Δχ2[1] = 0*.*46*, *p = 0*.*50)*	Not confirmed
H3c	Opportunities for interprofessional exchange, such as face- to face meetings are expected to contribute to better information sharing in both settings, but especially in specialized settings.	*(Δχ2[1] = 7*.*05*, *p<0*.*01)*	Confirmed

Note: * indicates p < 0.05

** indicates p < 0.01

*** indicates p < 0.001.

## Discussion

A vital aspect of quality of care in PC is the extent to which information is shared between HCPs who work together closely in a team. To optimize the quality of PC services provided, identifying organizational factors that enable explicit collaboration between coworkers is of utmost importance. Using a survey instrument, this study investigates the extent to which information transfer affects PC providers’ satisfaction with collaboration and, ultimately, their satisfaction with job-related tasks.

This paper contributes in two main ways to the existing literature on how information transfer is facilitated in the field of palliative care. First, we demonstrate the need for personal, face-to-face information exchange for PC providers who work in a team. Although it would be expected that electronic patient records in particular are essential for successful information sharing in the healthcare sector, this specific sample of palliative care providers highlights the fact that to date, opportunities for face-to-face meetings are paramount for successful information exchange in PC. Face-to-face meetings may be useful to support the social functions of healthcare teams, improving mutual respect in the care team, allowing team members to solve problems more efficiently, and facilitating the transmission of organizational culture [24]. In a study by Ellingston and colleagues, communication was reported to be the most effective in interdisciplinary healthcare teams, where both formal and informal meetings occurred on a regular basis [35].

Second, this research underlines that the success of interprofessional collaboration in PC is partially care-context dependent. This is due to the fact that primary and specialized care has evolved in isolation historically, with SPC showing higher levels of institutionalization and regulatory pathways for collaboration than PPC [27]. With regard to the aging population and growing burden of serious illness, SPC and PPC are both required to meet patients’ palliative care needs accordingly [36].

The results point to striking evidence that some organizational aspects affect successful information exchange between PC providers more drastically than others. Sufficient opportunities for face-to-face meetings and supervisions, feedback-tools to improve collaborative practices, and the application of guidelines and standards for collaboration were strong predictors for information exchange in PC teams. Based on our results, it is recommended that whenever institutes (hospitals, hospices, retirement homes, ambulant nursing organizations etc.) are establishing new collaborative processes in PC provision, they should aim to grant sufficient time for personal exchanges among the PC providers. Further, collaborative processes should be regularly evaluated in order to maintain and improve a sustainable social network between suppliers. Staff should be involved as early as possible in the improvement process to help ensure that changes correspond with their philosophy of collaboration [20].

Aligned with prior expectations, the study also found that colleagues who share the same work-related values reported significantly improved information transfer. High-functioning teams in healthcare settings should not only integrate principles of team-based care, but also agree on shared goals and values for delivery of patient-centered care [24]. Therefore, practice, healthcare facilities, GPs, and nursing organizations are encouraged to discuss and share their patient-centered values and ideals openly.

Furthermore, the study found considerable evidence that providers’ shared values, as well as their satisfaction with communication, positively predict their satisfaction with work-related tasks. This is a key finding, as the satisfaction of team members is linked to staff retention, which is a critical element for team functioning, as well as a predictor for good healthcare provision [24, 26].

The findings also suggest that frequent changes to caregiving negatively impact the information exchange in the team, as loss of information and misunderstandings occur easily when care teams are fluctuating. Much information is lost when health professionals change teams, therefore each PC team member should be trained to maintain the flow of information. Furthermore, making available written records of standards and guidelines on work procedures to all team members is recommended.

Unexpectedly, the study found little evidence that e-tools used to share patient files facilitate information transfer among PC team members. This is partly due to the fact that in Switzerland, e-tools for managing patient files are not yet mandatory for all healthcare providers and are far from being universally established [14]. However, in 2017, Switzerland passed a new federal law regarding patients’ electronic health records that requires hospitals and hospices to adopt interoperable electronic patient records by 2020 in order to facilitate information exchange among healthcare providers. Thus, future research in this area is needed once electronic patient records are introduced comprehensively in the sector of SPC and have further developed [14]. No relationships were found between the presence of a CM on the information exchange in PC teams. This finding requires further investigation, as CMs are not yet established across the board in PC, while representing a very inhomogeneous occupational group with differing job tasks [17]. Future research should investigate CMs’ possible effects on information transfer in certain facilities and care contexts.

This research contributes to a growing body of knowledge pointing to organizational differences between SPC and PPC, which are important to understand when considering future interventions to meet patients’ palliative care needs.

Given the diversity of organizational enablers for information transfer and collaboration presented above, we recommend further investigation into which variables affect information transfer while specifically distinguishing for PC teams in different care facilities and care contexts.

As with any research, we recognize the following limitations of our study. First, some of the dependent variables should be better operationalized in future research. This applies, for example, to the impact of CMs on information transfer in PC teams. Because the fields of activity and applications of CMs in palliative care in Switzerland remain largely unclear, we suggest that the role of CMs in palliative care be clarified in future investigations before definitive statements can be made about their impact on information transfer. The same caution applies to e-tools to share electronic patient files, which may depend on user characteristics, and user interface and user-friendliness; both of which contribute to successful communication in certain environments.

Perhaps the main limitation of this study is that it lacks the attributes of a standardized questionnaire to assess information transfer and organizational aspects of care. Future studies are advised to use the Care Process Self-Evaluation tool (CPSET), as seen in in the work of Seys and colleagues [37]. However, both the questionnaire used in this study and the CPSET tool are based on self-evaluation by healthcare professionals, which may contribute to bias. The results of this study may be further biased due to the use of a convenience sample of PC providers who volunteered to participate in the online survey. This can lead to a selection bias in the sense that study participants might be more engaged in PC than average and thus rate organizational aspects of care provision differently. In this study, certain professional groups were only assessed marginally (e.g. psychologists, social workers, physiotherapists); thus, representing a dimension that could be expanded upon in future PC research. Furthermore, the study findings are based upon solely Swiss PC providers and therefore was not attempting to be representative of other countries. For future scientific research endeavors, it would be of great value to replicate our study in different healthcare settings, or even with transnational data.

## Conclusion

Particularly in the field of palliative care, institutions, employers, and other stakeholders, such as the federal administrations, desire to be informed about organizational factors that improve the exchange of information between PC providers. The present study is intended to serve as a basis for recommendations as to how information transfer and communication can be improved by the establishment of certain organizational enablers in interdisciplinary PC teams.

## Supporting information

S1 File Questionnaire in German. (DOCX)

S2 File Questionnaire translated to English. (DOCX)
