# Supplementary material for: Organizational determinants of information transfer in palliative care teams: A structural equation modeling approach
Source: PLoS One. 2021 Jun 3;16(6):e0252637. doi: 10.1371/journal.pone.0252637 (PMC8174710; doi:10.1371/journal.pone.0252637)
Supplement: S1 File — (DOCX) [file pone.0252637.s001.docx]

**Fragebogen zu Organisatorischen Einflussfaktoren auf Informationstransfer in Palliative-Care Netzwerken**

1. **Geschlecht:  m  w**

**2. Bitte geben Sie Ihren Jahrgang an: ___________**

**3. Bitte geben Sie Ihre höchste Ausbildung an:**

 Obligatorische Schulzeit (mind. 9 Jahre)

 Eidg. Berufsattest / Lehrabschluss / eidg. Fähigkeitszeugnis

 Maturität (Gymnasial, Berufs- oder Fachmaturität)

 Eidg. Fachausweis / höhere Fachschule / eidg. Diplom

 Bachelor (FH, Universität, ETH)

 Master (FH, Universität, ETH)

 PhD / Doktorat / Dr. med.

 anderes, nämlich: ____________________________

**4. Bitte geben Sie Ihre jetzige Funktion im Bereich der Palliativversorgung an:**

 Hausarzt/ Hausärztin (Dr. med., Med. pract.)

 Spezialisierter Arzt / Ärztin (Dr. med., Med. pract.)

 AssistentIn Gesundheit und Soziales AGS EBA (früher PflegeassistentIn (PA) oder Spitalgehilfe/-in)

 Fachfrau/-mann Betreuung EFZ (FaBe) (früher: BetagtenbetreuerIn (FA SODK))

 Fachfrau/-mann Gesundheit EFZ (FaGe) (früher: Fachangestellte/-r Gesundheit, Praktische Krankenpflege (PKP) FA SRK)

 Pflegefachfrau/-mann Diplomniveau I

 Dipl. Pflegefachfrau/-mann HF  Dipl. Pflegefachfrau/-mann (BScN, FH)

 Dipl. Pflegefachfrau/-mann (MScN)

 Advanced Practice Nurse (APN)

 PhysiotherapeutIn / ErgotherapeutIn

 PsychologIn

 Kunst-, Tier-, oder MusiktherapeutIn

 Pastorale Dienste / SeelsorgerInnen

**** SozialarbeiterIn

 ApothekerIn / Pharma-AssistentIn

 Case ManagerIn

 Freiwillige/r

**** anderes, nämlich: ____________________________

**5. Bitte geben Sie die Einwohnerzahl Ihres Arbeitsortes an:**

** Grossstadt** ≥ 100‘000 EW (Zürich, Genf, Basel, Bern, Lausanne und Winterthur)

** grössere Stadt** ≥ 20‘000 und < 100‘000 EW

** Kleinstadt** oder Kleinstadt im städtischen Einzugsgebiet ≥ 10‘000 und < 20‘000 EW

** Agglomeration** bzw. Dorf im städtischen Einzugsgebiet < 10‘000 EW

** ländliche Region / Dorf** oder **Bergregion** < 10‘000 EW

**6. Besitzen Sie eine Zusatzausbildung im Bereich Palliative Care?**

 ja  nein

**7. Gibt es eine klare Verantwortungsteilung in Ihrem Bereich bzw. in Ihrer Institution, wenn es um die Palliativversorgung geht?**

|  ja |  eher ja |  eher nein |  nein |  weiss nicht |
| --- | --- | --- | --- | --- |

**8. Gibt es in ihrem unmittelbaren Arbeitsumfeld häufige Wechsel der primären Betreuungs-personen von Patientinnen?**

 ja  eher ja  eher nein  nein  weiss nicht

**9. Die Kommunikation innerhalb unserer Organisation / unseres Institutes ist gut.**

 Trifft völlig zu  Trifft zu  Trifft eher zu  Trifft eher nicht zu  Trifft nicht zu  Trifft überhaupt nicht zu

**10. Ich mag die Hauptaufgaben, die meine Arbeit im Bereich der Palliativversorgung umfasst.**

 Trifft völlig zu  Trifft zu  Trifft eher zu  Trifft eher nicht zu  Trifft nicht zu  Trifft überhaupt nicht zu

**11. Personen, mit denen ich täglich in der Palliativversorgung zusammenarbeite, teilen meine Werte und Ideale bezüglich Palliative Care.**

 Trifft völlig zu  Trifft zu  Trifft eher zu  Trifft eher nicht zu  Trifft nicht zu  Trifft überhaupt nicht zu

**12. Gibt es im Rahmen Ihrer unmittelbaren Zusammenarbeit eine/n Case ManagerIn?**

 Ja  Nein Weiss nicht

**13. Benutzen Sie bzw. Ihre Institution in Ihrer Zusammenarbeit ein Formular zur Rückmeldung (Feedback-Tool), um die Wünsche und Bedürfnisse von PalliativpatientInnen zu erheben?**

 Ja  Nein Weiss nicht

**14. Benutzen Sie an Ihrem Arbeitsort elektronische Mittel (E-Tools) zur Verwaltung von Patientendossiers?**

 Ja  Nein Weiss nicht

**15. Stehen Ihnen und Ihrem Arbeitsumfeld interne Guidelines und Standards für die Palliativversorgung zur Verfügung?**

 Ja  Nein Weiss nicht

**16. Falls ja: Finden diese Guidelines Ihrer Erfahrung nach Anwendung in Ihrem Arbeitsalltag?**

 Ja  Nein Weiss nicht

**17. Werden Arbeitsabläufe in Ihrer Organisation / Praxis / Ihrem unmittelbaren Arbeitsumfeld regelmässig evaluiert, z.B. in Form von Qualitätszirkeln oder Feedbackrunden?**

 Ja  Nein Weiss nicht

**18. Wie beurteilen Sie im Hinblick auf Ihr unmittelbares Arbeitsumfeld: den Informationsaustausch zwischen den Personen, mit denen Sie für die Betreuung von PalliativpatientInnen verantwortlich sind.**

 Sehr gut  Gut  Genügend  Ungenügend Schlecht  Sehr schlecht

**19. Wie beurteilen Sie im Hinblick auf Ihr unmittelbares Arbeitsumfeld…die Möglichkeit zum interprofessionellen Austausch in Ihrer Organisation / Praxis (z.B. im Rahmen von Meetings, Supervisionen, Qualitätszirkeln etc.).**

 Sehr gut  Gut  Genügend  Ungenügend Schlecht  Sehr schlecht
