# Supplementary material for: Organizational determinants of information transfer in palliative care teams: A structural equation modeling approach
Source: PLoS One. 2021 Jun 3;16(6):e0252637. doi: 10.1371/journal.pone.0252637 (PMC8174710; doi:10.1371/journal.pone.0252637)
Supplement: S2 File — (DOCX) [file pone.0252637.s002.docx]

**Organizational factors that influence information transfer in palliative care networks**

1. Please indicate your sex:  m  f

2. Please indicate your date of birth (DD/MM/YY): _____________

3. Please indicate your highest education:

- Compulsory education (at least 9 years)
- Federal professional certificate / apprenticeship
- Matura (grammar school, vocational or specialist school-leaving certificate)
- Federal professional card / higher technical college / federal diploma
- Bachelor (University of Applied Sciences or Arts, University, ETH)
- Master (University of Applied Sciences or Arts, University, ETH)
- PhD / Doctorate / Dr. med.
- other, namely: ____________________________

4. Please indicate your current function in palliative care:

- Family physician/ general practitioner
- Specialist doctor
- Assistant for Health and Social Services (formerly nursing assistant or hospital assistant)
- Specialist in elderly care
- Health specialist EFZ (FaGe) (formerly: health specialist nurse)
- Nurses Diploma level I
- Dipl. nurse with a Bachelor of Science
- Dipl. nurse with a Master of Science
- Advanced Practice Nurse (APN)
- Physiotherapist / Occupational therapist
- Psychologist
- Art, animal or music therapist
- Pastoral carer
- Social worker
- Pharmacist / Pharmaceutical Assistant
- Case Manager
- Volunteer
- another, namely: ____________________________

5. Please indicate the population of your place of work:

- Large city ≥ 100'000 inhabitants (Zurich, Geneva, Basel, Bern, Lausanne and Winterthur)
- Larger city ≥ 20'000 and < 100'000 p.e.
- Small town or town in urban catchment area ≥ 10'000 and < 20'000 p.e.
- Agglomeration or village in urban catchment area < 10'000 p.e.
- Rural region / village or mountain region < 10'000 p.e.

6. Do you have additional training in palliative care?

- yes
- no

7. Do you have a clear division of responsibilities in your area or institution when it comes to palliative care?

- yes
- rather yes
- rather no
- no
- do not know

8. Are there frequent changes in the primary caregivers of female patients in their immediate working environment?

- yes
- rather yes
- rather no
- no
- do not know

9. The communication within our organization / institute is good:

- Applies completely
- Rather applies
- Rather not applies
- Applies not at all

10. I like the main tasks that my work in palliative care involves.

- Applies completely
- Rather applies
- Rather not applies
- Applies not at all

11. People with whom I work daily in palliative care share my values and ideals regarding palliative care

- Fully agree
- Agree
- Rather agree
- Rather don`t agree
- Disagree
- Fully disagree

12. Is there a case manager in your direct cooperation?

- Yes
- No
- Don`t know

14. Do you use electronic means (e-tools) at your place of work to manage patient files?

- Yes
- No
- Don`t know

15. Are internal guidelines and standards for palliative care available to you and your work environment?

- Yes
- No
- Don`t know

16. if so, in your experience, are these guidelines applied in your everyday work?

- Yes
- No
- Don`t know

17. are work processes in your organisation / practice / immediate working environment regularly evaluated, e.g. in the form of quality circles or feedback rounds?

- Yes
- No
- Don`t know

18. Please think about your immediate working environment: how do you assess the exchange of information between the people with whom you are responsible for the care of palliative patients?

- Very good
- Good
- Sufficient
- Insufficient
- Poor
- Very poor

19. How do you rate the possibilities for inter-professional exchange in your organization / practice (e.g. in the context of meetings, supervision, quality circles etc.)

- Very good
- Good
- Sufficient
- Insufficient
- Poor
- Very poor
